# Minimally invasive repair of iatrogenic bile duct injuries: a systematic review and meta-analysis

**DOI:** 10.1007/s00464-026-12715-7

**Published:** 2026-03-30

**Authors:** Marios Alogakos, Ahmed Ghani, Abdullah R. Ayesh, May Y. Hajeir, Lamees M. Al Darwashi, Christian A. Than, Hayato Nakanishi, Benjamin E. Johnson, Alexandra M. Roch, Eugene P. Ceppa

**Affiliations:** 1 Department of Surgery, Division of Surgical Oncology, Indiana University School of Medicine, 545 Barnhill Drive, EH 541, Indianapolis, IN 46202 USA; 2 School of Medicine, University College Dublin, Dublin, D04 C1P1 Ireland; 3 School of Medicine, University of Nicosia, 2417 Nicosia, Cyprus; 4 Cambridge University Hospitals NHS Foundation Trust, Addenbrookes Hospital, Cambridge, CB2 0QQ UK; 5 School of Biomedical Sciences, The University of Queensland, St Lucia, 4072 Australia; 6 Department of Surgery, Mayo Clinic, Rochester, MN 55905 USA; 7 Department of Surgery, Endeavor Health, Chicago, IL 60625 USA

**Keywords:** Bile duct injury, Laparoscopic, Robotic, Minimally invasive, Surgery, Meta-analysis

## Abstract

**Background:**

Bile duct injuries (BDI) are a rare but serious complication of cholecystectomy, associated with substantial morbidity and clinical burden. However, no consensus has been reached on the optimal surgical approach for the management of BDI. This meta-analysis aims to evaluate the safety and efficacy of minimally invasive surgery (MIS) for the repair of iatrogenic BDI.

**Methods:**

Cochrane, CINAHL, Ovid Embase and Medline were searched from their inception date to March 2025. The Preferred Reporting Items for Systematic Reviews and Meta-Analysis reporting guidelines were followed. The review was registered prospectively on PROSPERO (CRD420251009530).

**Results:**

A total of 246 patients with MIS for BDI repair from sixteen studies were included in the analysis. The pooled mean operative time was 219 min (95% CI: 183–255), and the pooled mean hospital stay was 6.5 days (95% CI: 5.2–7.8). The pooled mean estimated blood loss was 122 mL (95% CI: 84–160). The overall postoperative morbidity was 16.2% (95% CI: 0.092–0.233, I^2^ = 60%, *n* = 47). The rate of biliary fistula was 4.8% (95% CI: 0.021–0.075, I^2^ = 0%, *n* = 13), while the rate of stricture was 3.9% (95% CI: 0.015–0.063, I^2^ = 0%, *n* = 9). The rate of conversion to open surgery was 2.3% (95% CI: 0.002–0.044, I^2^ = 0%, *n* = 4), while the rate of readmission was 4.5% (95% CI: 0.003–0.086, I^2^ = 0%, *n* = 7). The reoperation rate was 3.0% (95% CI: 0.009–0.052, I^2^ = 0%, *n* = 8) and there was no surgery-associated mortality across the included studies.

**Conclusions:**

MIS appears safe and effective for the repair of iatrogenic BDIs and is associated with acceptable morbidity and low reoperation rate. However, given the lack of high-level evidence, further studies are needed to ascertain these findings.

**Graphical abstract:**

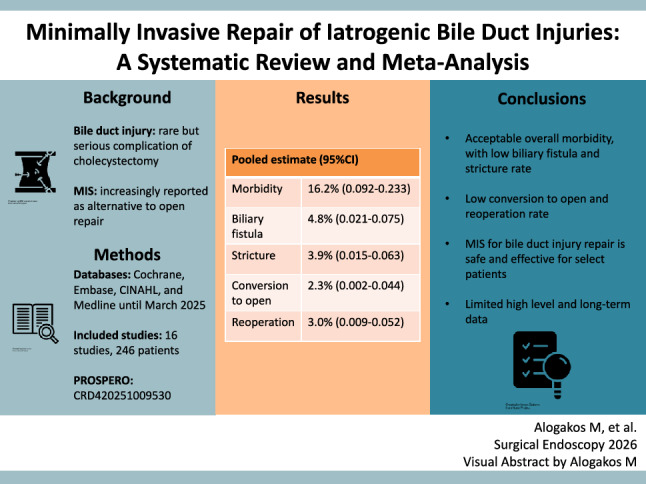

**Supplementary Information:**

The online version contains supplementary material available at 10.1007/s00464-026-12715-7.

Bile duct injury (BDI) is one of the most serious morbidities that may arise during general surgical procedures, often stemming from an expected highly variant biliary anatomy or misidentification of critical structures due to hindered visualization secondary to severe inflammation [1, 2]. The majority of reported BDIs occur during laparoscopic cholecystectomy, with an incidence of 0.3–0.7%, and rarely during other hepato-pancreato-biliary (HPB) procedures [3]. With more than 750,000 cholecystectomies performed annually, making it the most common general surgery procedure in the US, the morbidity associated with BDIs constitutes a substantial clinical and financial burden [3–7]. While prevention remains the ultimate goal, intraoperative detection of BDIs or timely diagnosis and management by HPB surgeons is critical in limiting adverse sequelae and allowing for optimal repair [1, 8–10].

The primary goal of surgical repair of BDI is to mitigate both short- and long-term complications and to ensure an uneventful recovery [11]. Furthermore, the cornerstone of surgical treatment, regardless of technique, involves a tension-free bilioenteric anastomosis that ensures optimal alignment of mucosal tissue with adequate ductal perfusion [1, 12]. Traditionally, an open approach has been favored for more severe BDIs, owing to the need for a more precise bilioenteric anastomosis [13, 14]. However, more recently, minimally invasive surgery (MIS) has emerged as a promising alternative for BDI repair [8, 13].

Despite numerous studies showing encouraging results with minimally invasive BDI repairs, a consensus has yet to be reached regarding its potential efficacy [1]. For instance, Goméz et al. investigated BDI repair through laparoscopic Roux-en-Y hepaticojejunostomy (RYHJ) and reported similar clinical outcomes to open repair [15]. Additionally, Sucandy et al. reported lower intraoperative estimated blood loss (EBL) and intensive care unit (ICU) admission rates in patients undergoing robotic BDI repair compared with an open approach [16]. Notably, a recent meta-analysis by the Society of American Gastrointestinal and Endoscopic Surgeons and American and the Americas Hepato-Pancreato-Biliary Association (SAGES-AHPBA) identified only a single study directly comparing MIS and open repair, underscoring the profound scarcity of comparative data and the very low certainty of available evidence in this field [17].

With the current lack of consensus, as acknowledged by the World Society of Emergency Surgery (WSES) and the SAGES guidelines, questions regarding the role of MIS in risk assessments, surgical planning, and patient selection criteria have been raised in the context of repairing BDIs [1, 17]. Moreover, to the best of our knowledge, no existing meta-analysis has evaluated perioperative outcomes in patients undergoing minimally invasive repair in the setting of BDI. Thus, this study aims to investigate the safety and efficacy of MIS in the repair of iatrogenic BDI.

## Materials and methods

### Search strategy and data sources

A comprehensive search of Cochrane, CINAHL, Ovid Embase and Medline from each database’s inception to March 10th, 2025, was conducted. This review was reported following the Preferred Reporting Items for Systematic Reviews and Meta-Analysis (PRISMA) 2020 reporting standards [18]. The search strategy was designed and conducted by an experienced librarian in collaboration with the study’s principal investigator. Controlled vocabulary supplemented with keywords was used to identify adult patients undergoing MIS for BDI repair. A detailed description of all search terms is available in Supplementary Item 1. The review was registered prospectively with PROSPERO (CRD420251009530).

### Eligibility criteria

Randomized controlled trials (RCTs), cohort studies, case–control studies, or case series that met the following inclusion criteria were considered eligible for inclusion: 1) Adult participants ≥ 18 years of age who underwent MIS for iatrogenic BDI; 2) Outcomes of overall postoperative morbidity; and/or 3) Reoperation rates. Case reports, abstracts, reviews, conference abstracts, and articles that were not reported in English were excluded. Additionally, studies reporting on open surgery as the initial approach were excluded. The eligibility of studies was assessed based on intention-to-treat analysis.

### Screening and risk of bias assessment

The risk of bias for each study was independently evaluated by two authors (LMD and MYH) using the Joanna Briggs Institute (JBI) Critical Appraisal Checklist for Case Series [19]. Studies were considered to have low risk of bias if at least 8 items were rated “Yes”, moderate risk if 5 to 7 items were rated “Yes”, and high risk if fewer than 5 items were rated “Yes”. Any discrepancies were discussed by the two independent assessors, with disagreements addressed via an adjudicator (MA). Two out of five independent assessors (AG, ARA, LMD, MA, MYH) conducted individual article screening and data extraction. Any disagreements were discussed with co-authors and adjudicated by HN. The certainty of evidence was evaluated using the Grading of Recommendations, Assessment, Development, and Evaluation (GRADE) approach, with priori thresholds set for small, moderate, and large effects [20].

### Extracted outcomes

**Fig. 1:**
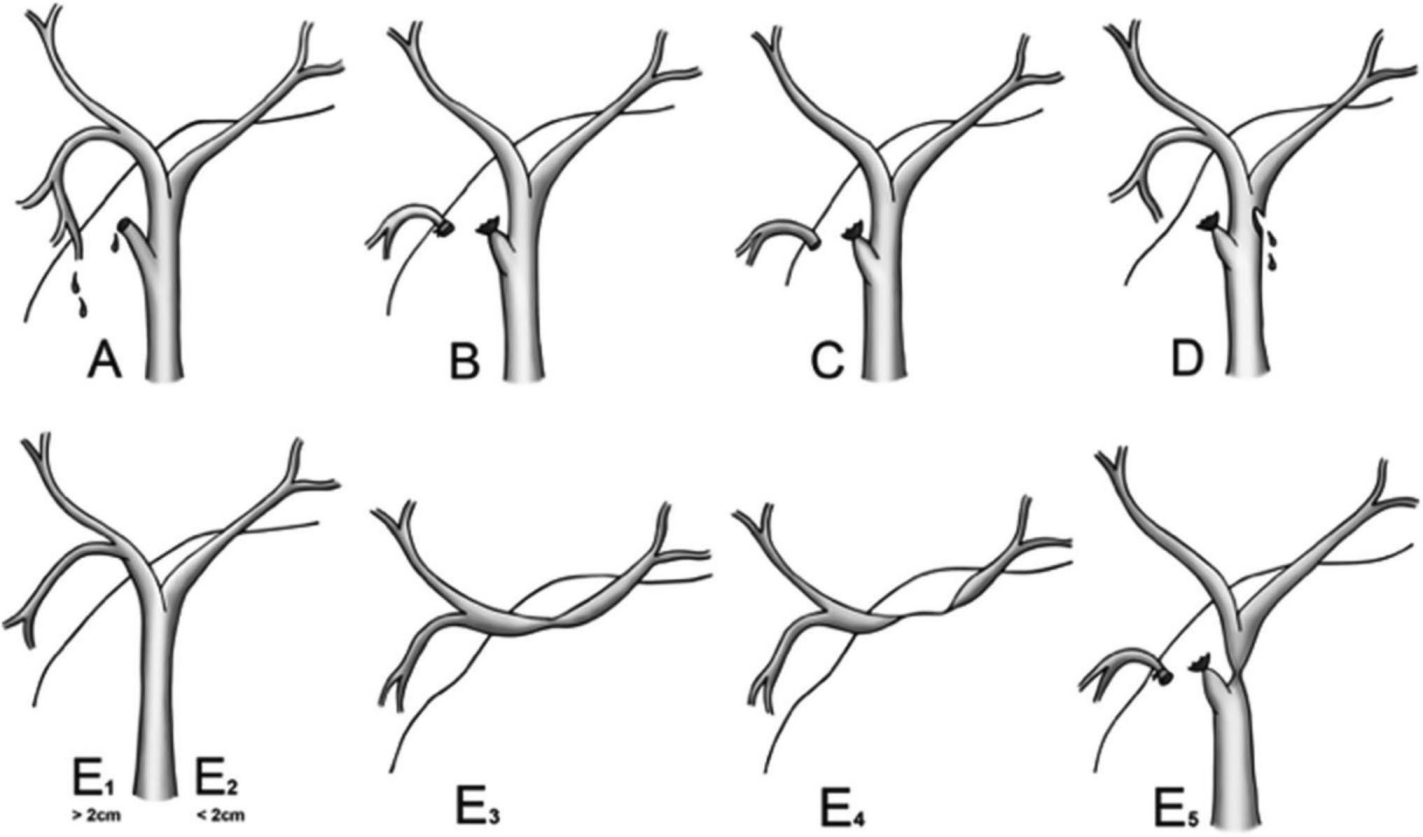
The Bismuth-Strasberg classification of bile duct injuries. **A** Bile leak from cystic duct or liver bed, **B** Occlusion of an aberrant right hepatic duct, **C** Bile leak from an aberrant right hepatic duct, **D** Lateral injury to the common bile duct (< 50% circumference), **E1** Common bile duct injury, > 2 cm from the confluence, **E2** Common bile duct injury, < 2 cm from the confluence, **E3** Hilar injury with preserved confluence, **E4** Hilar injury with involvement of confluence, **E5** Injury of aberrant right sectoral duct and common bile duct

This analysis aimed to evaluate the outcomes of MIS in the repair of iatrogenic BDI. The baseline characteristics extracted were as follows: presenting symptoms, American Society of Anesthesiologists (ASA) score, body mass index (BMI), time from injury to repair, Bismuth-Strasberg classification **(**Fig. 1**)**, cause of injury, and institution of repair (same as index operation or referral institution) [21]. The following perioperative outcomes were extracted: operative approach and technique, operative time, EBL, length of hospital stay, and morbidity. For postoperative outcomes, the following were extracted: postoperative nothing per oral (NPO) time, readmission, recurrence and reoperation rates, rate of conversion to open approach, and mortality rates.

### MIS and surgical technique selection criteria

The selection criteria for MIS and surgical technique were rarely reported and varied across studies [9, 10, 22–24]. Minimally invasive repair was considered in patients with an intact biliary confluence and no associated vascular injury [22], and it was avoided when local dissection was deemed unsafe [23]. In another study, MIS was selectively performed in patients with high-output fistulas or large intra-abdominal fluid collections, provided there was no jaundice or suspicion of complete bile duct transection [10]. Pekolj et al. utilized MIS when there was limited thermal injury, and the surgeon was experienced in bile duct exploration and intracorporeal suturing techniques [9]. Regarding the selection of surgical technique, a laparoscopic hepaticoduodenostomy (HD) was preferred in Bismuth class I–III injuries with minimal fibrosis around the duodenum, whereas RYHJ was favored in cases involving friable ducts, multiple ductal openings, a non-pliable duodenum, or early repair [24].

### Statistical analysis

The pooled means and proportions were analyzed using the random-effects, generic inverse variance method of DerSimonian and Laird, which assigns the weight of each study based on its variance [25]. Subgroup analyses were performed based on surgical approach (robotic and laparoscopic) and injury Bismuth-Strasberg classification (D & E1-E5). The heterogeneity of effect size estimates across the studies was quantified using the Q statistic and I^2^. A value of I^2^ of 0–25% indicates insignificant statistical heterogeneity, 26–50% low heterogeneity, and 51–100% high heterogeneity [26]. Furthermore, a leave-one-out sensitivity analysis was conducted to assess each study’s influence on the pooled estimate by omitting one study at a time and recalculating the combined estimates for the remaining studies. The median was converted to mean using the formulas from the Cochrane Handbook for Systematic Reviews of Interventions, when the mean was unavailable [27]. Data analysis was performed using Open Meta analyst software (CEBM, Brown University, Providence, Rhode Island, USA).

## Results

### Study selection and patient characteristics

The initial literature search yielded 5502 potentially relevant articles, of which sixteen unique studies [9, 10, 14–16, 22–24, 28–35] involving 246 patients were included in this analysis. The selected studies included thirteen retrospective cohort studies [9, 10, 14, 15, 23, 24, 28, 29, 31–35], two case series [22, 30], and one prospective cohort study [16]. Fifteen studies were conducted in a single center, and one was multicenter [34]. The reported mean age ranged from 38 to 55 years, and 69.7% (n = 161) of patients were female. Four studies [14, 16, 29, 31] reported the preoperative BMI with a pooled mean of 28.2 kg/m^2^ (95% CI: 25.1–31.3, I^2^ = 87%). A PRISMA flowchart of the study selection process is depicted in Supplementary Item 2. The baseline characteristics of the included studies are described in Table 1.

**Table 1:** Baseline characteristics of included studies

Study	Publication (Year)	Country	Study type	Number of centers (n)	Total participants (n)	Gender (Female), n (%)	Age, mean ± SD (Years)	BMI mean ± SD (kg/m2)	Length of follow-up, mean ± SD (Months)
Barband et al	2011	Iran	Retrospective	Single center	10	NR	NR	NR	NR
Chen et al	2013	China	Retrospective	Single center	3	1 (33)	NR	NR	36.0 ± 0
Chowbey et al	2005	India	Retrospective	Single center	4	3 (75)	38.3 ± 9.5	NR	NR
Cuendis-Velazquez et al	2019	Mexico	Retrospective	Single center	75	57 (76)	43.1 ± 15.7	25.3 ± 3.1	28.0 ± 26.7
D’Hondt et al	2023	Belgium	Case series	Single center	4	3 (75)	54.8 ± 10.8	NR	NR
Dokmak et al	2017	France	Case series	Single center	3	2 (67)	NR	NR	NR
Giulianotti et al	2018	US	Retrospective	Single center	14	12 (86)	45.9 ± 23.4	32.1 ± 6.2	36.1 ± 28.1
Gomez et al	2020	Colombia	Retrospective	Single center	20	14 (70)	49.0 ± 6.5	NR	NR
Javed et al	2021	India	Retrospective	Single center	29	23 (79)	40.0 ± NR	NR	9.0 ± 3.0
Kwak et al	2019	Korea	Retrospective	Single center	8	6 (75)	48.3 ± 12.0	NR	36.4 ± 11.8
Kwon et al	2001	Japan	Retrospective	Single center	11	7 (64)	48.0 ± 14.3	NR	NR
Marino et al	2019	Italy	Retrospective	Single center	12	8 (67)	55.0 ± 10.3	28.3 ± 3.6	12.2 ± 7.5
Pekolj et al	2013	Argentina	Retrospective	Single center	5	NR	NR	NR	NR
Sahoo et al	2022	India	Retrospective	Multicenter	16	7 (44)	46.0 ± 8.8	NR	28.0 ± 9.0
Sucandy et al	2022	US	Prospective	Single center	8	5 (63)	50.0 ± 16.3	28.0 ± 7.4	22.0 ± 9.5
Tantia et al	2008	India	Retrospective	Single center	24	13 (54)	51.5 ± 8.0	NR	11.2 ± 8.3

*BMI* Body mass index, *n* Sample size, *NR* Not reported, *SD* Standard deviation, *US* United States

### Risk of bias assessment and certainty of evidence

The results of the critical appraisal of all included studies are shown in Supplementary Item 3. Twelve studies [9, 10, 14, 15, 22, 24, 29, 31–35] were judged to have low risk of bias, while four studies [16, 23, 28, 30] were judged to have moderate risk of bias due to unclear inclusion criteria and a lack of appropriate adjustment for confounding factors. Nonetheless, all the studies included were deemed adequate within the selection domain. The results of the certainty of evidence assessment are summarized in a GRADE evidence table in Supplementary Item 4.

### Clinical characteristics

**Fig. 2:**
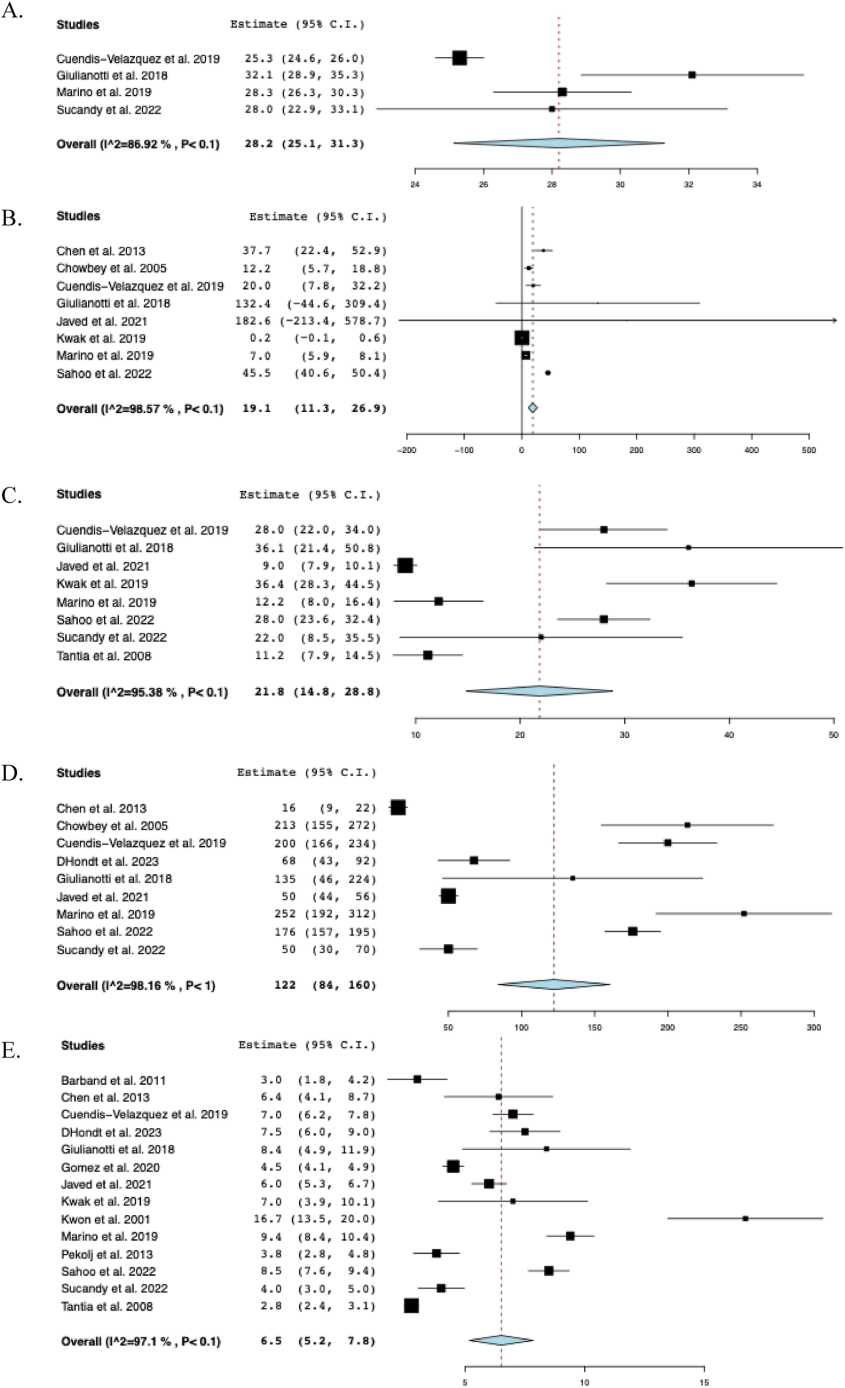

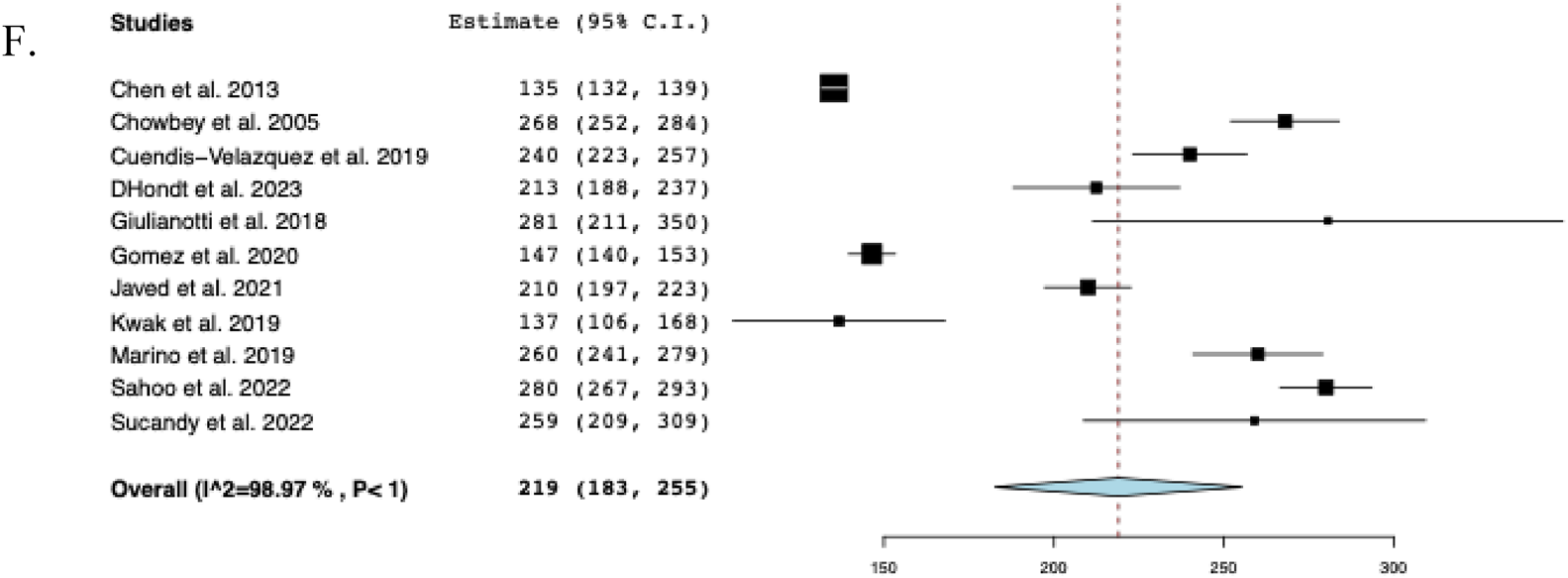
The pooled clinical characteristics: **A** Preoperative body mass index, **B** Time from injury to repair, **C** Follow-up, **D** Estimated blood loss, **E** Hospital Stay, **F** Operative time

The most common clinical presentations associated with iatrogenic BDI across nine studies [10, 14, 22–24, 28–30, 34] were the following: jaundice (50.6%, *n* = 79), bilious drainage secondary to fistula (26.9%, *n* = 42), cholangitis (26.3%, *n* = 41), and peritonitis (13.4%, n = 21). The mean preoperative ASA score was 1.9 ± 3.1 in two studies [14, 16]. Thirteen studies [10, 14, 15, 22–24, 28, 29, 31–35] reported the cause of BDI, most commonly following laparoscopic cholecystectomy (69.7%, *n* = 159), followed by open cholecystectomy (22.8%, *n* = 52) and laparoscopic cholecystectomy converted to open (7.5%, *n* = 17). Across nine studies [14, 15, 22, 24, 29, 31, 32, 34, 35], the rate of preoperative endoscopic retrograde cholangiopancreatography (ERCP) usage was 51.2% (*n* = 103). The pooled mean follow-up period after BDI repair across eight studies [14, 16, 24, 29, 31, 32, 34, 35] was 21.8 months (95% CI: 14.8–28.8, I^2^ = 95%), with the longest follow-up period being 36.4 months [32]. The clinical characteristics of the included studies are summarized in Table 2 and Fig. 2.

**Table 2:** Clinical characteristics of included studies

Study	Surgical approach (n)	Bismuth-Strasberg classification (n)									Repair technique (n)			
		A	B	C	D	E1	E2	E3	E4	E5	RYHD	RYHJ	Primary repair	Kasai
Barband et al	L	NR	NR	NR	NR	NR	NR	NR	NR	NR	0	0	10	0
Chen et al	L	NR	NR	NR	NR	NR	NR	NR	NR	NR	0	3 **	0	0
Chowbey et al	L	NR	NR	NR	NR	NR	NR	NR	NR	NR	0	4	0	0
Cuendis-Velazquez et al	L (40) R (35)	0	0	0	0	8	14	31	19	3	0	75	0	0
D’Hondt et al	R	0	0	0	0	0	2	0	1	0	0	4	0	0
Dokmak et al	L	NR	NR	NR	NR	NR	NR	NR	NR	NR	0	3	0	0
Giulianotti et al	R	0	0	0	0	0	6	5	2	0	0	12	0	2
Gomez et al	L	0	0	0	0	13	4	2	1	0	0	20	0	0
Javed et al	L	0	0	0	0	4	3	13	5	4	16	13	0	0
Kwak et al	L	0	0	0	0	3	2	0	2	1	0	0	8	0
Kwon et al	L	NR	NR	NR	NR	NR	NR	NR	NR	NR	0	0	11	0
Marino et al	R	0	0	0	0	2	8	1	1	0	0	12	0	0
Pekolj et al	L	NR	NR	NR	NR	NR	NR	NR	NR	NR	0	0	5	0
Sahoo et al	L	0	0	0	0	16	0	0	0	0	0	16	0	0
Sucandy et al	R	NR	NR	NR	NR	NR	NR	NR	NR	NR	1 *	7	0	0
Tantia et al	L	11	0	4	9	0	0	0	0	0	2 *	0	22	0

*L* Laparoscopic, *n* Sample size, *NR* Not reported, *R* Robotic, *HD* Hepaticoduodenostomy, *RYHJ* Roux−en−Y hepaticojejunostomy, *SD* Standard deviation

^*^: Choledochoduodenostomy

^**^: Roux−en−Y cholangiojejunostomy

### Injury and repair characteristics

Across ten studies [14, 15, 22–24, 29, 31, 32, 34, 35], the BDI was recognized intraoperatively (38.2%, *n* = 78) or postoperatively (61.8%, *n* = 126). Among fourteen studies [10, 14, 15, 22–24, 28–35], the repair was performed at the same time of injury in 39 cases (16.7%). The mean time from iatrogenic BDI to repair was 19.1 days (95% CI: 11.3–26.9, I^2^ = 99%) across nine studies [14, 23, 24, 28, 29, 31, 32, 34, 35]. Six studies [14, 24, 31, 32, 34, 35] reported the site of repair, which consisted of the same institution as the injury (49.5%, n = 51) or a referral center (50.5%, n = 52). Fifteen studies [10, 14–16, 22–24, 28–35] reported the operative approach, consisting of laparoscopic (70.3%, n = 173) and robotic surgery (29.7%, *n* = 73). Nine studies [14, 15, 24, 29–32, 34, 35] reported the Bismuth-Strasberg classification which was the following: A (5.5%, *n* = 11), C (2.0%, *n* = 4), D (4.5%, *n* = 9), E1 (23.0%, *n* = 46), E2 (19.5%, *n* = 39), E3 (26.0%, *n* = 52), E4 (15.5%, *n* = 31), and E5 (4.0%, *n* = 8). Across the sixteen studies, the most common operative technique was Roux-en-Y hepatico/cholangiojejunostomy (68.7%, *n* = 169). This included RYHJ (*n* = 166) and Roux-en-Y cholangiojejunostomy (RYCJ) (*n* = 3). RYHJ was further subclassified into Hepp-Couinaud-like reconstruction (n = 53), neo-confluence (*n* = 23), and RY-bi-HJ (*n* = 2). Hepatico/choledochoduodenostomy accounted for 7.7% (n = 19), consisting of HD (*n* = 16) and choledochoduodenostomy (*n* = 3). Other techniques included primary repair (22.8%, n = 56) and the Kasai procedure (0.8%, *n* = 2). The injury and repair characteristics of the included studies are comprehensively described in Table 2.

### Postoperative outcomes

**Fig. 3:**
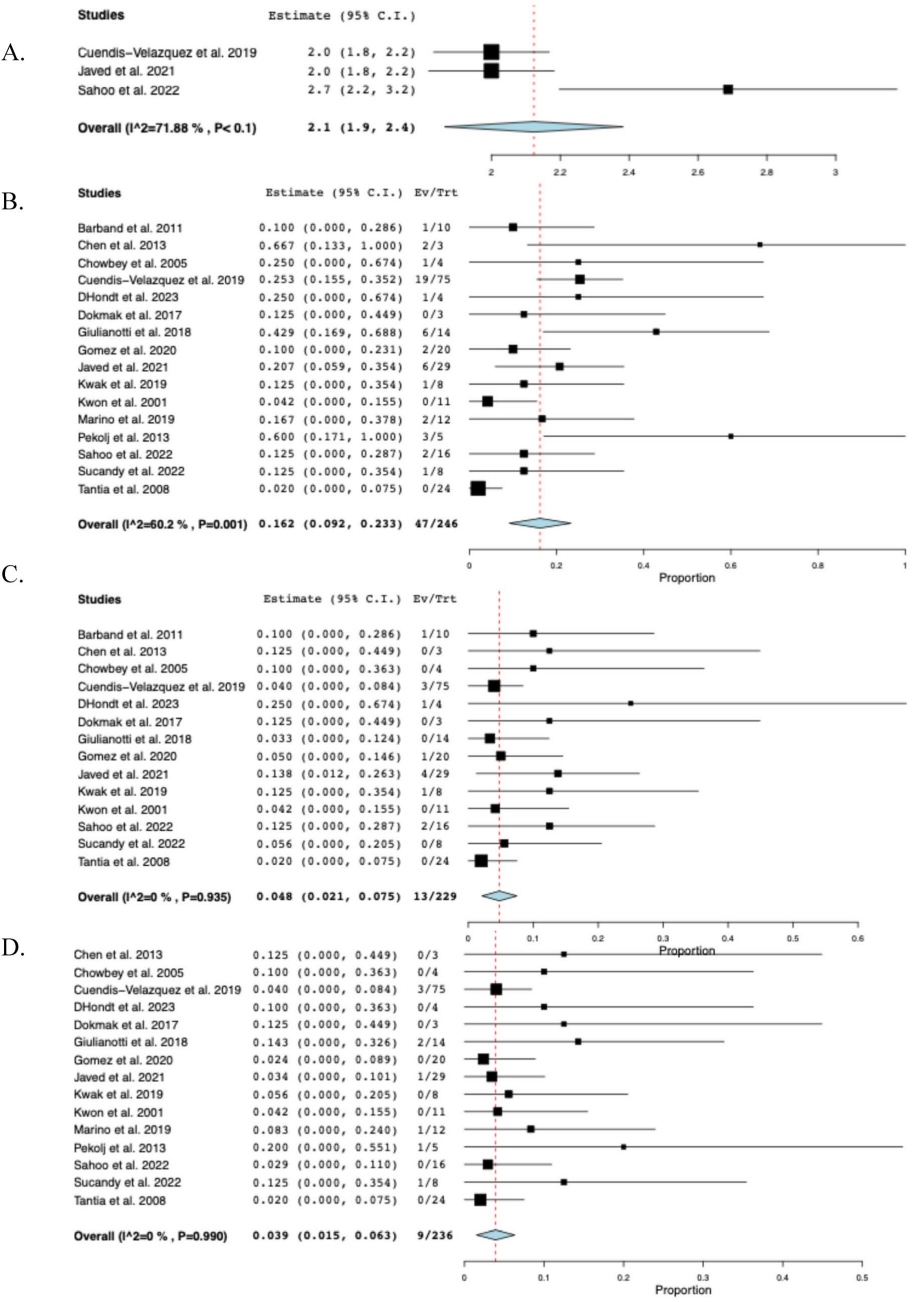

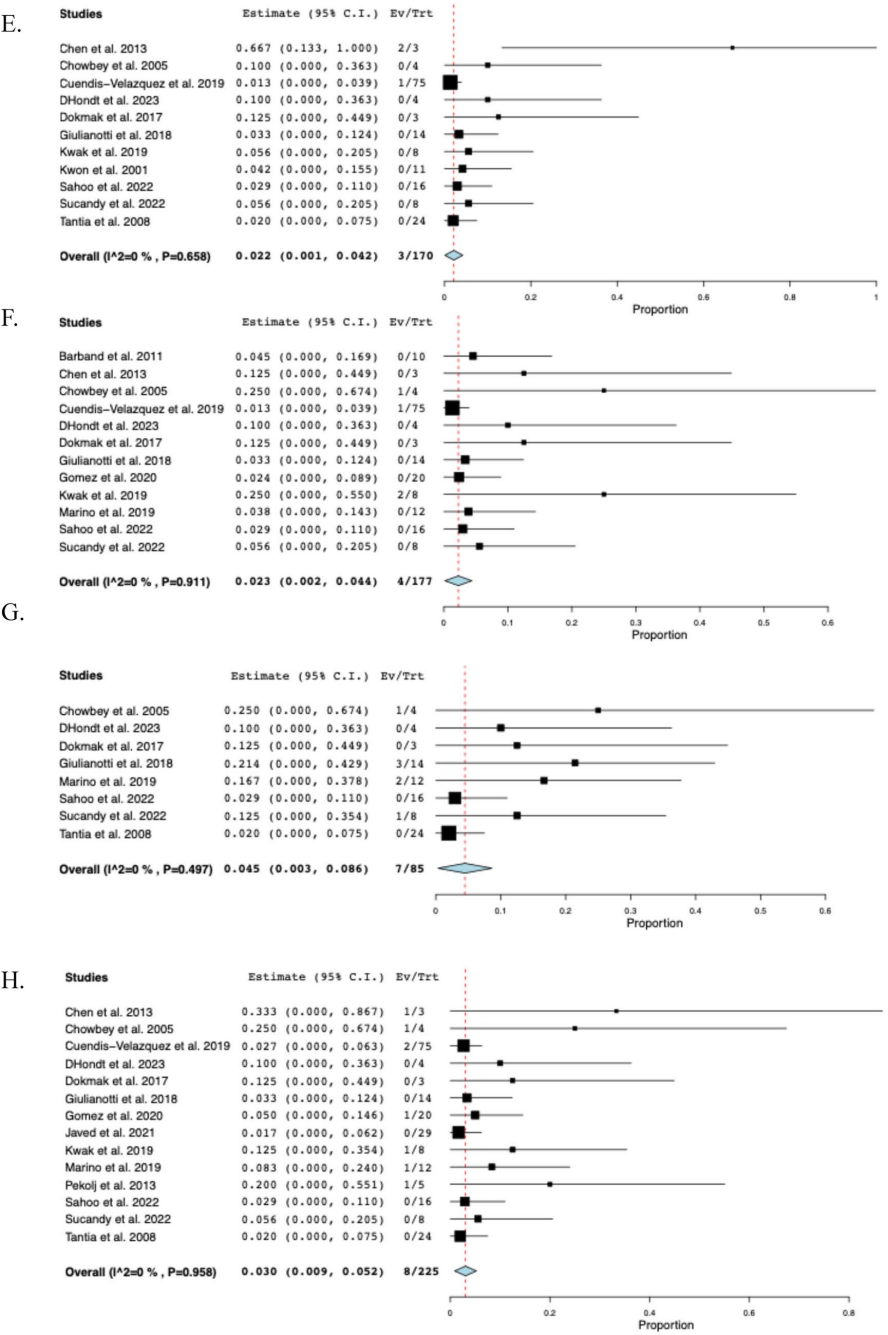
The pooled postoperative outcomes. **A** Postoperative nothing per oral time, **B** Morbidity, **C** Biliary fistula, **D** Stricture, **E** Hematoma, **F** Conversion to open approach, **G** Readmission, **H** Reoperation

The pooled mean operative time was 219 min (95% CI: 183–255, I^2^ = 99%) across eleven studies [14–16, 23, 24, 28–32, 34], and the pooled mean hospital stay was 6.5 days (95% CI: 5.2–7.8, I^2^ = 97%) across fourteen studies [9, 10, 14–16, 23, 24, 29–35]. Nine studies [14, 16, 23, 24, 28–31, 34] reported EBL with a reported pooled mean of 122 mL (95% CI: 84–160, I^2^ = 98%). Across three studies [24, 29, 34], the mean postoperative NPO time was 2.1 days (95% CI: 1.9–2.4, I^2^ = 72%). In sixteen studies, overall postoperative morbidity was 16.2% (95% CI: 0.092–0.233, I^2^ = 60%, n = 47). Across fourteen studies [10, 15, 16, 22–24, 28–35], the rate of biliary fistula was 4.8% (95% CI: 0.021–0.075, I^2^ = 0%, *n* = 13), while the rate of stricture was 3.9% (95% CI: 0.015–0.063, I^2^ = 0%, n = 9) across fifteen studies [9, 14–16, 22–24, 28–35]. The timing of stricture ranged from 35 days to 18 months postoperatively, with only 2 (22%) occurring more than 1 year following BDI repair. Among eleven studies [16, 22, 23, 28–35], the rate of hematoma formation was 2.2% (95% CI: 0.001–0.042, I^2^ = 0%, n = 3). Across twelve studies [10, 14–16, 22, 23, 28–32, 34], the rate of conversion to open surgery was 2.3% (95% CI: 0.002–0.044, I^2^ = 0%, n = 4). The reasons for conversion were described as follows: considerable anatomic distortion and fibrosis (n = 2), difficulty in controlling bleeding at the gallbladder bed (n = 1), and severe inflammation due to bile leak (n = 1). Among eight studies [14, 16, 22, 28, 30, 31, 34, 35], the rate of readmission was 4.5% (95% CI: 0.003–0.086, I^2^ = 0%, n = 7), while the reoperation rate among fourteen studies [9, 14–16, 22–24, 28–32, 34, 35] was 3.0% (95% CI: 0.009–0.052, I^2^ = 0%, n = 8). Across five studies [9, 23, 28, 29, 32], reoperation was required due to intra-peritoneal hemorrhage (n = 1), HJ leak (n = 3) within 3 months postoperatively and biliary stricture (n = 3) at 1, 3 and 18 months postoperatively. There were two unrelated deaths at 21 and 90 days postoperatively, secondary to acute myeloid leukemia and pancreatic cancer, respectively [29, 32]. The perioperative outcomes of included studies are comprehensively described in Table 3 and Fig. 3.

**Table 3:** Perioperative and postoperative outcomes of included studies

Study	Operative time mean ± SD (min)	Hospital stay mean ± SD (days)	EBL mean ± SD (mL)	Biliary fistula (n)	Stricture (n)	Hematoma (n)	Morbidity (n)	Reoperation (n)	Conversion to open approach (n)
Barband et al	NR	3.0 ± 2.0	NR	1	NR	NR	1	NR	0
Chen et al	135 ± 3	6.4 ± 2.0	16 ± 6	0	0	2	2	1	0
Chowbey et al	268 ± 16	4.0 ± 0	213 ± 60	0	0	0	1	1	1
Cuendis-Velazquez et al	240 ± 74	7.0 ± 3.7	200 ± 148	3	3	1	19	2	1
D’Hondt et al	213 ± 25	7.5 ± 1.5	68 ± 25	1	0	0	1	0	0
Dokmak et al	NR	NR	NR	0	0	0	0	0	0
Giulianotti et al	281 ± 132	8.4 ± 6.7	135 ± 170	0	2	0	6	0	0
Gomez et al	147 ± 16	4.5 ± 1.0	NR	1	0	NR	2	1	0
Javed et al	210 ± 35	6.0 ± 2.0	50 ± 18	4	1	NR	6	0	NR
Kwak et al	137 ± 45	7.0 ± 4.5	NR	1	0	0	1	1	2
Kwon et al	NR	16.7 ± 4.5	NR	0	0	0	0	NR	NR
Marino et al	260 ± 34	9.4 ± 1.8	252 ± 106	NR	1	NR	2	1	0
Pekolj et al	NR	3.8 ± 1.1	NR	NR	1	NR	3	1	NR
Sahoo et al	280 ± 28	8.5 ± 1.8	176 ± 39	2	0	0	2	0	0
Sucandy et al	259 ± 73	4.0 ± 1.4	50 ± 28	0	1	0	1	0	0
Tantia et al	NR	2.8 ± 0.8	NR	0	0	0	0	0	NR

*EBL* Estimated blood loss, *n* Sample size, *NR* Not reported, *SD* Standard deviation

### Subgroup analysis based on surgical approach: robotic and laparoscopic

**Fig. 4:**
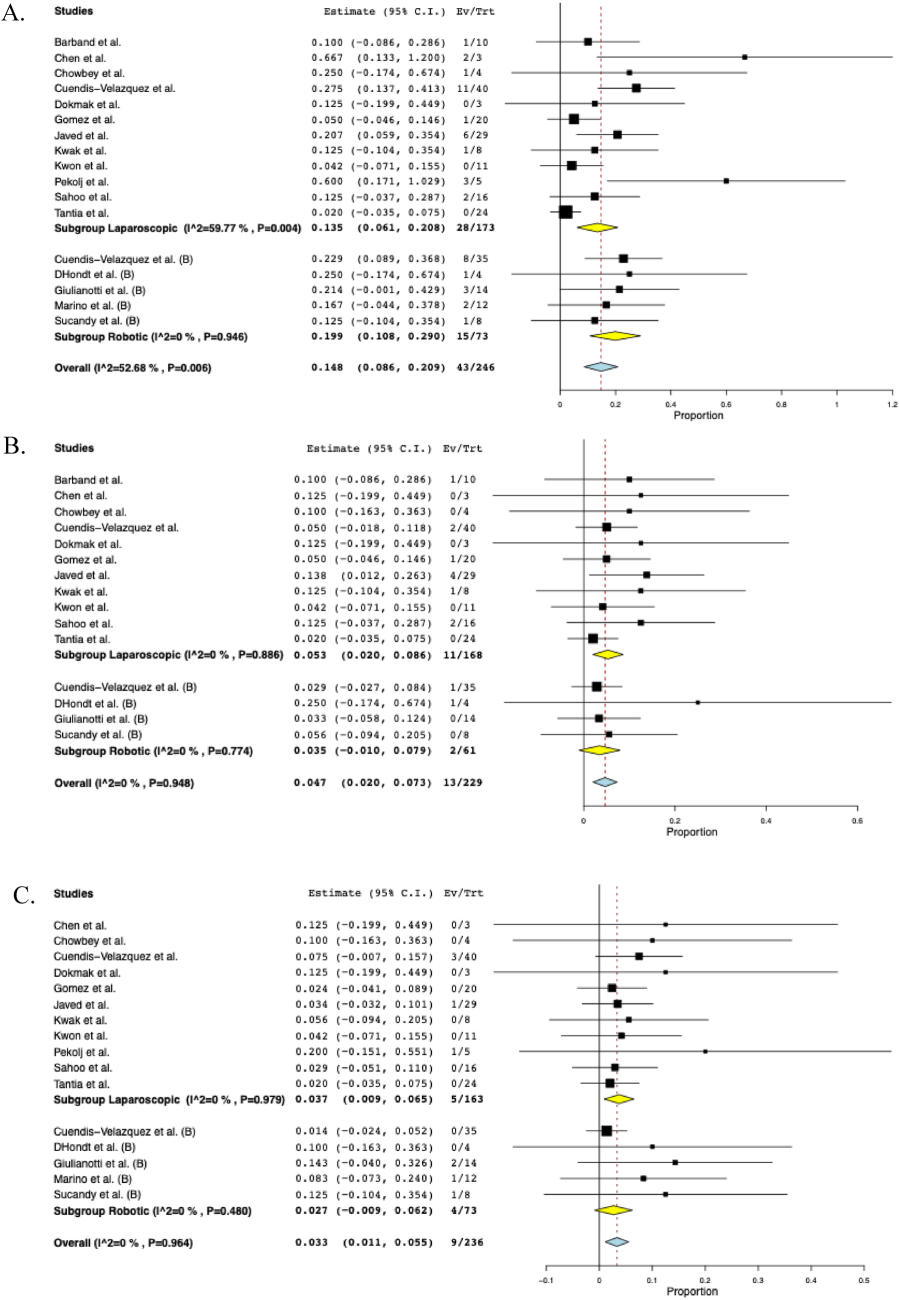

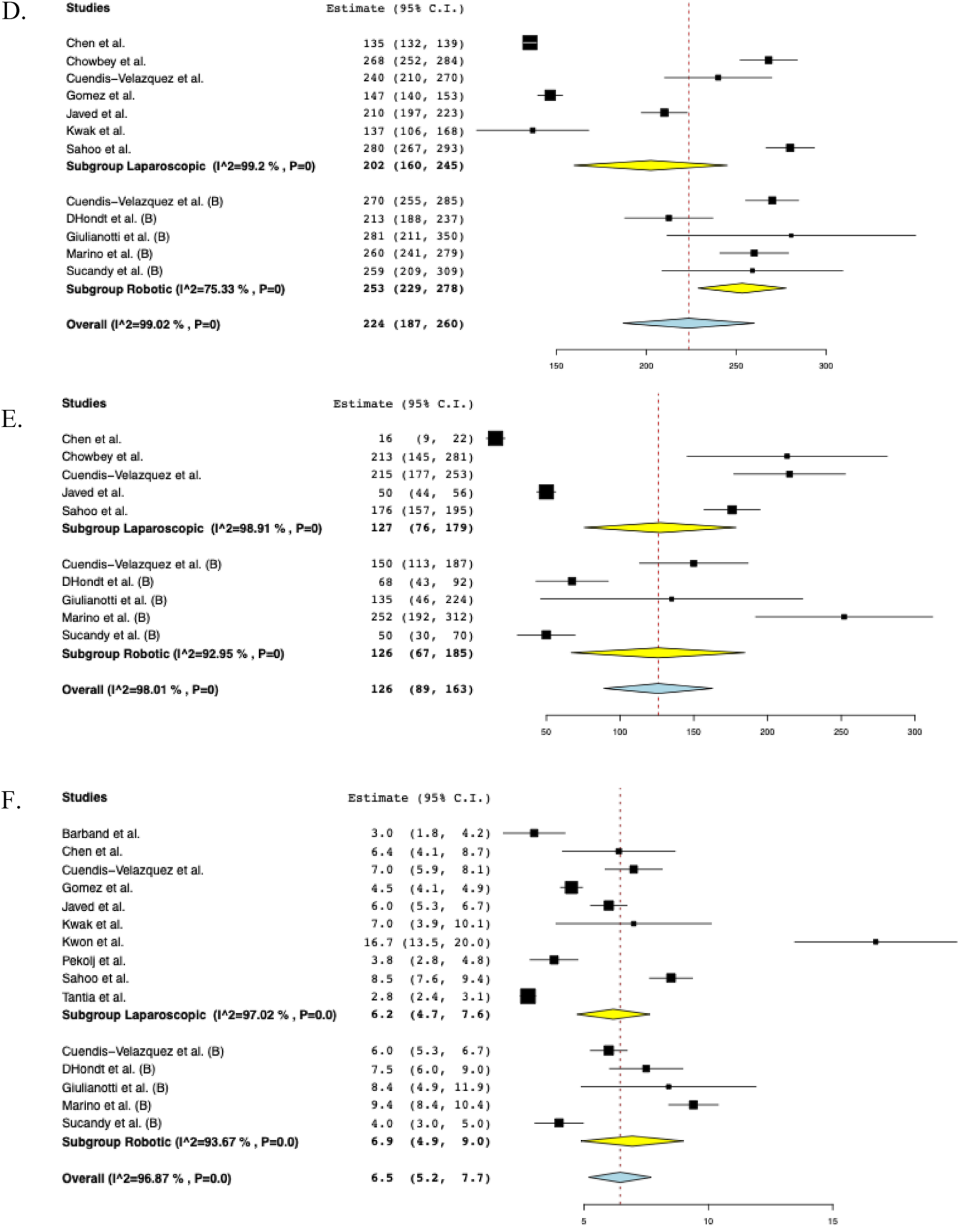

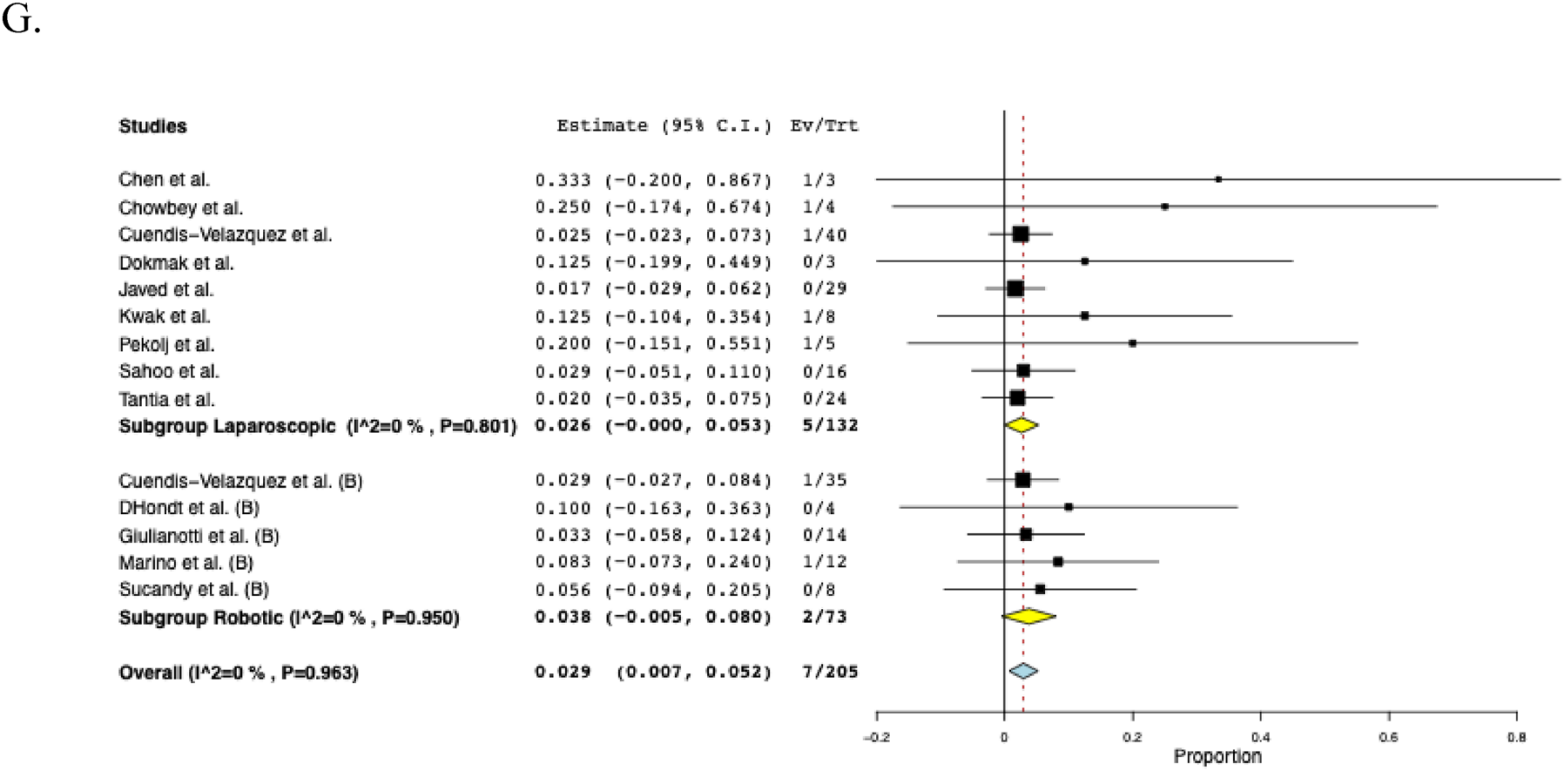
The pooled estimate postoperative outcomes of subgroup analysis based on surgical approach. **A** Morbidity, **B** Biliary fistula, **C** Stricture, **D** Operative time, **E** Estimated blood loss, **F** Hospital stay, **G** Reoperation

A subgroup analysis was performed based on the surgical approach, including laparoscopic (*n* = 173) and robotic (*n* = 73). The overall postoperative morbidity was 13.5% (95% CI: 0.061–0.208, I^2^ = 60%, *n* = 28) for the laparoscopic cohort and 19.9% (95% CI: 0.108–0.290, I^2^ = 0%, *n* = 15) for the robotic cohort. The rate of biliary fistula was 5.3% (95% CI: 0.020–0.086, I^2^ = 0%, *n* = 11) for the laparoscopic group and 3.5% (95% CI: -0.010–0.079, I^2^ = 0%, *n* = 2) for the robotic group. The rate of stricture was 3.7% (95% CI: 0.009–0.065, I^2^ = 0%, *n* = 5) for patients undergoing laparoscopic surgery and 2.7% (95% CI: -0.009–0.062, I^2^ = 0%, *n* = 4) for patients undergoing robotic surgery. The pooled operative time was 202 min (95% CI: 160–245, I^2^ = 99%) in the laparoscopic group and 253 min (95% CI: 229–278, I^2^ = 75%) in the robotic group. EBL was similar between groups, with a pooled mean of 127 mL (95% CI: 76–179, I^2^ = 99%) in the laparoscopic group and 126 mL (95% CI: 67–185, I^2^ = 93%) in the robotic group. The length of hospital stay was 6.2 days in the laparoscopic group (95% CI: 4.7–7.6, I^2^ = 97%) compared with 6.9 days in the robotic cohort (95% CI: 4.9–9.0, I^2^ = 94%). The reoperation rate was 2.6% (95% CI: -0.000–0.053, I^2^ = 0%, *n* = 5) in the laparoscopic cohort and 3.8% (95%CI: 0.005–0.080, I^2^ = 0%, *n* = 2) in the robotic cohort. The subgroup analyses based on surgical approach are depicted in Fig. 4.

### Subgroup analysis solely including Bismuth-Strasberg class D & E1-E5

**Fig. 5:**
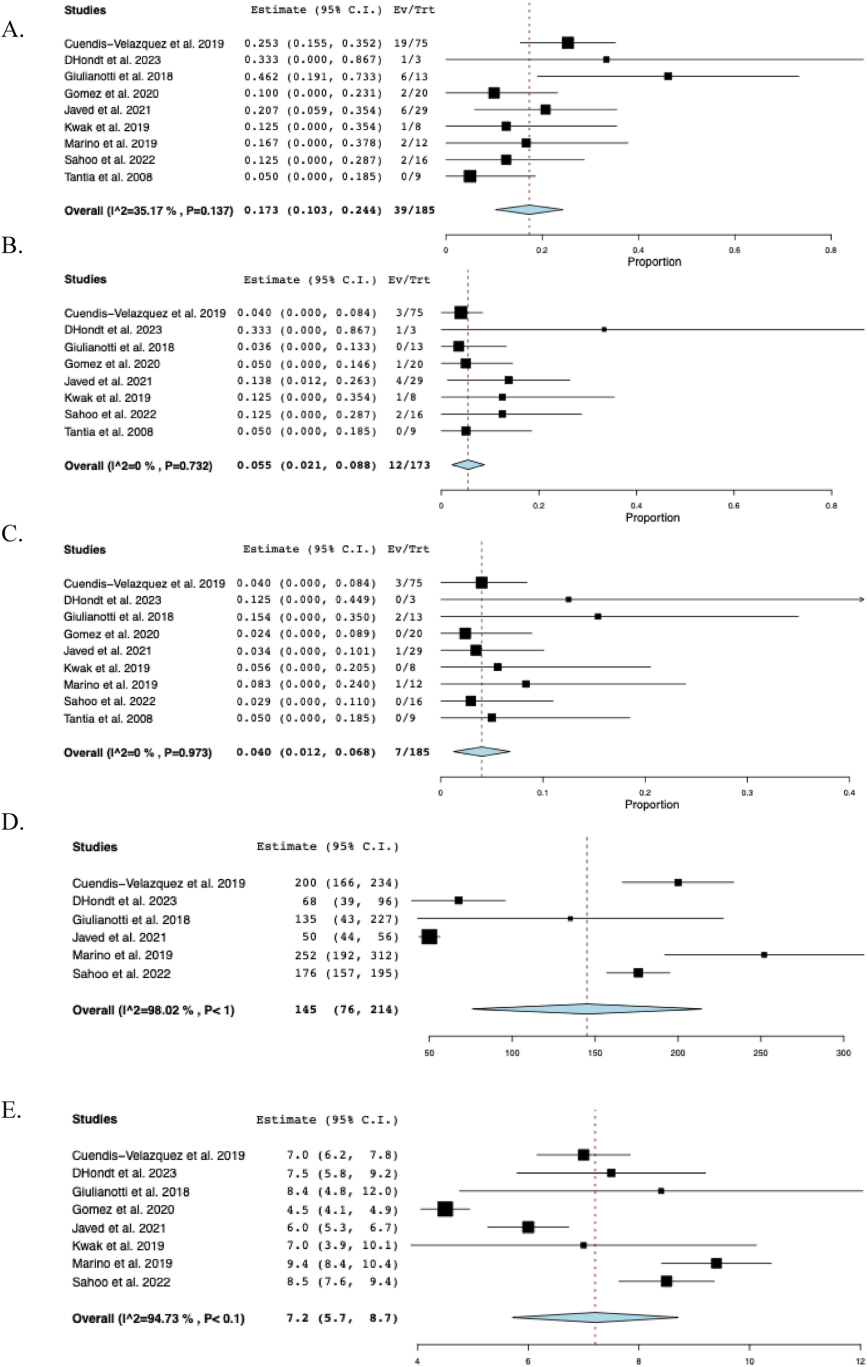

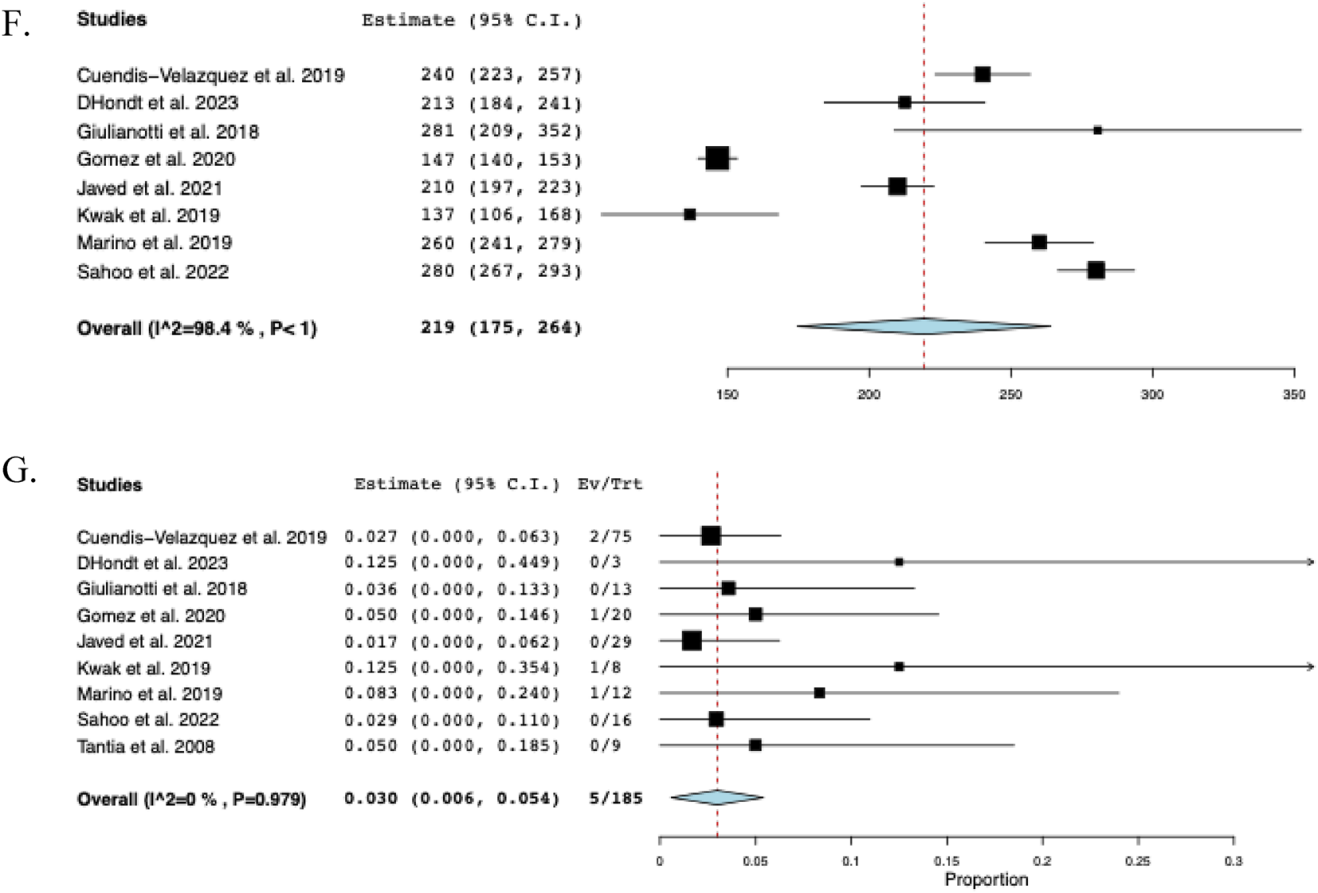
The pooled estimate postoperative outcomes of subgroup analysis including Bismuth-Strasberg class D & E1-E5. **A** Morbidity, **B** Biliary fistula, **C** Stricture, **D** Estimated blood loss, **E** Hospital stay, **F** Operative time, **G** Reoperation

A further subgroup analysis was performed, only including injuries classified as Bismuth-Strasberg D or E1–E5. These consisted of the following: D (*n* = 9), E1 (*n* = 46), E2 (*n* = 39), E3 (*n* = 52), E4 (*n* = 31), and E5 (*n* = 8). The subgroup postoperative morbidity was 17.3% (95% CI: 0.103–0.244, I^2^ = 35%, *n* = 39). The rate of biliary fistula was 5.5% (95% CI: 0.021–0.088, I^2^ = 0%, *n* = 12) while the rate of stricture was 4.0% (95% CI: 0.012–0.068, I^2^ = 0%, *n* = 7). EBL was 145 mL (95% CI: 76–214, I^2^ = 98%). The length of hospital stay was 7.2 days (95% CI: 5.7–8.7, I^2^ = 95%). The pooled operative time was 219 min (95% CI: 175–264, I^2^ = 98%). The reoperation rate was 3.0% (95% CI: 0.006–0.054, I^2^ = 0%, *n* = 5). The subgroup analyses on Bismuth-Strasberg class D & E1–E5 are shown in Fig. 5.

## Discussion

Iatrogenic BDI remains one of the most feared morbidities of cholecystectomy. The incidence of major BDI, historically reported at 0.1% to 0.2% in the era of open cholecystectomy, increased to approximately 0.3% to 0.6% with the widespread adoption of the laparoscopy, with a recent study reporting even higher rates with the robotic approach in the elderly population [36, 37]. No guidelines have been established regarding the optimal approach for BDI repair at the time of this study, yet open repair had been the historic approach with reproducible and high-quality outcomes. Accordingly, this meta-analysis aimed to evaluate the safety and efficacy of MIS for BDI repair. Based on sixteen included studies, our pooled analysis demonstrated an acceptable overall morbidity rate and low rates of major morbidity, including biliary fistula and stricture. Our subgroup analysis demonstrated that the laparoscopic approach was associated with lower morbidity and operative time compared to robotic surgery.

Our meta-analysis provides the most comprehensive pooled estimates to date for minimally invasive BDI repair, showing a biliary fistula rate of 4.8%. This is a substantial improvement compared with early laparoscopic reports documenting biliary fistula rates higher than 19%, which raised concerns regarding the safety and durability of minimally invasive repair [38]. Various forces likely inspired this improvement. First, the standardization of techniques at HPB centers emphasized that wide, tension-free hepaticojejunostomy, with precise mucosa-to-mucosa apposition and avoidance of ischemia, facilitates fewer fistulas and more durable patency. Refinements in MIS techniques coupled with wider adoption and increased surgeon experience have likely contributed to these improved outcomes [39]. Furthermore, advances in MIS platforms, particularly robotic surgery, have been critical. Conventional laparoscopy is hindered by the fulcrum effect, 2D imaging, and rigid instruments, making delicate anastomoses technically more challenging [40]. Notably, the robotic platform overcomes some of these limitations by providing stable 3D visualization, tremor filtration, and wristed instruments with a greater range of motion. These features facilitate more precise intracorporeal suturing that enables a consistent creation of a meticulous, tension-free anastomosis. This is fundamental to a durable biliary reconstruction which may plausibly explain a decrease in the observation of biliary fistulas in MIS repair [41]. However, while contemporary series suggest evolution in operative practice, the heterogeneity and limited granularity of reported technical details preclude establishing a direct association between specific operative modifications and the observed improvements in outcomes. Our findings underscore that contemporary minimally invasive repair can achieve biliary fistula outcomes that are similar to open repair series [42].

Consistent with our findings, robotic-assisted repair had demonstrated low stricture rates with minimal conversions to open surgery. Despite these favorable outcomes, the widespread adoption of robotic-assisted repair remains limited by several factors [14, 41]. Current platforms lack true haptic feedback, which may hinder fine tissue handling, although upcoming platforms such as Da Vinci series 5 aim to address this limitation. Beyond technical aspects, broader adoption is constrained by high cost, steep learning curves, and concentration of expertise in specialized centers [43]. Importantly, the latest SAGES guidelines confirm that minimally invasive repairs do not pose higher risks when performed in appropriately selected cases [17]. More specifically, they may be preferred in stable patients with favorable anatomy or with obesity, where MIS can enhance exposure and visualization, whereas open repair remains appropriate for unstable patients or those with complex, high injuries or severe inflammation.

Our pooled analysis also demonstrated stricture rates of 3.9%, which was lower than historical series reporting rates of 10–20%, over a median time of 11–30 months [1, 38, 44, 45]. This underscores that improvements in minimally invasive repair extend beyond early outcomes and translate into durable long-term patency. Such durability is likely related to the lower incidence of intra-abdominal infection and bile peritonitis with contemporary MIS techniques, which reduces the chronic inflammatory response that drives anastomotic fibrosis and late strictures [1, 46]. Timing of reconstruction was also critical. Repairs carried out early (< 2 weeks) or delayed (> 6 weeks) once inflammation has resolved were consistently associated with better outcomes. In contrast, attempts in the intermediate 2- to 6-week window often faced higher risks of fistula and stricture, as tissue was friable, edematous and prone to poor healing [47, 48]. Surgeon expertise and centralization further strengthened outcomes. When the first repair was performed by HPB surgeons, patency rates were consistently higher than after attempts by non-specialists, reflecting not just greater technical skill, but also perioperative decision-making such as the routine use of RYHJ over historical end-to-end repairs, which carried higher failure rates [49–51]. Specialist centers also prioritized preoperative optimization, ensuring adequate drainage, infection control, and high-quality imaging which supported long-term success [52]. Finally, these findings align with the SAGES guidelines which emphasized that vigilant postoperative surveillance remained critical, as even biliary fistulas detected within the first month postoperatively may predispose to future narrowing if not promptly addressed [17].

In our pooled cohort, RYHJ was by far the predominant reconstruction technique. While the included studies did not provide direct comparative outcomes between different techniques, their importance is well recognized as a key determinant of both short‐ and long-term outcomes [39]. Direct comparative studies of RYHJ and HD were lacking, yet Cuendis-Velazquez et al. reported a morbidity rate of 23.3% following RYHJ whereas Moraca et al. demonstrated excellent long-term biliary function for both HD and RYHJ [41, 53]. RYHJ remained the preferred option across most high-volume HPB centers, often offering a well-vascularized, tension-free anastomosis and demonstrating long-term patency rates exceeding 90% in experienced hands [39, 41, 53, 54]. However, HD may be advantageous in select patients with localized injuries, minimal inflammation, and favorable anatomy as it preserved physiological bile flow into the duodenum. Moreover, HD was technically less complex because it required a single anastomosis, and facilitated endoscopic access for postoperative cholangiography or biliary intervention [53]. Regardless of the type of reconstruction chosen, meticulous technical execution, ensuring precise mucosa-to-mucosa approximation, and creation of an adequately wide anastomotic lumen, remain critical for minimizing stricture and fistula rates [55]. In principle, MIS provides better ergonomics, reduced tissue trauma, reduced pain, quicker recovery and comparable morbidity [8, 13]. Given the scarcity of granular technical reporting across the current literature, stratified documentation of operative outcomes based on the surgical technique is essential for future research, as it would minimize the substantial proportion of the variability observed in current outcomes [13].

This meta-analysis does not come without limitations. First, the majority of included studies were retrospective in design, potentially introducing inherent biases such as selection bias, information bias, and uncontrolled confounding. Second, there was substantial heterogeneity across studies in terms of injury severity, surgical expertise, institutional protocols, and case volume. Third, stratified data of MIS based on timing of repair (early vs. delayed repair) were not reported uniformly across studies, preventing any meaningful subgroup analysis to assess their impact on outcomes. Fourth, while both laparoscopic and robotic approaches were included under the umbrella of MIS, comparative data were not available, thus only allowing for indirect comparison of proportions in the subgroup analysis. Fifth, differences in surgeon experience, availability of technology, and standardized indications were not consistently reported and might have contributed to outcome variability. In addition, the low collective sample size limits the power of these findings. The rate of conversion to open surgery should be interpreted with caution given the reported conversion rates may underestimate the true incidence, as immediate conversions at the time of injury recognition are often not classified as conversions and thus excluded. Long-term endpoints such as anastomotic patency and late stricture recurrence were either inconsistently reported or assessed over highly variable follow-up durations, limiting the ability to make robust conclusions about long-term repair durability. Furthermore, variability in how overall morbidity and biliary fistula were defined and measured across studies may have influenced pooled estimates, thus caution is required when interpreting these results. Finally, the possibility of publication bias must be considered. Underreporting of complications, particularly in lower-volume centers or early in a surgeon’s experience with minimally invasive techniques, may have skewed outcomes. Moving forward, future studies should aim to standardize reporting of BDI characteristics, operative details, and follow-up protocols. Stratification by surgical approach, repair timing, and injury classification remains essential to allow for more meaningful comparisons and generate high-quality evidence guiding clinical practice.

## Conclusion

This review demonstrates that MIS for BDI repair achieves encouraging outcomes, with favorable morbidity and low rates of biliary fistula, stricture, and reoperation. Although the certainty of the evidence is low to moderate, the results suggest that MIS can be considered a safe and effective option in experienced hands for select patients. However, given the significant burden of BDIs, open repair currently remains the most established reconstruction approach. Despite these promising results, further studies with standardized reporting, higher case volume, and longer follow-up periods are needed to optimize patient selection and confirm the long-term durability of the repair.

## Supplementary Information

Below is the link to the electronic supplementary material.

Supplementary file1 (TIFF 5480 KB)

Supplementary file1 (TIFF 270 KB)

Supplementary file1 (TIFF 3656 KB)

Supplementary file1 (TIFF 241 KB)

## Data Availability

The data set used for this meta-analysis will be shared upon request from the study authors.

## Abbreviations

AHPBA: Americas hepato-pancreato-biliary association
ASA: American Society of Anesthesiologists
BDI: Bile duct injury
BMI: Body mass index
CI: Confidence interval
EBL: Estimated blood loss
ERCP: Endoscopic retrograde cholangiopancreatography
GRADE: Grading of recommendations, assessment, development, and evaluation
HD: Hepaticoduodenostomy
HPB: Hepato-pancreato-biliary
ICU: Intensive care unit
JBI: Joanna Briggs institute
MIS: Minimally invasive surgery
PRISMA: Preferred reporting items for systematic reviews and meta-analysis
RYCJ: Roux-en-Y choledochojejunostomy
RYHJ: Roux-en-Y Hepaticojejunostomy
SAGES: Society of American gastrointestinal and endoscopic surgeons
WSES: World society of emergency surgery
