# Correction: Minimally invasive repair of iatrogenic bile duct injuries: a systematic review and meta-analysis

**DOI:** 10.1007/s00464-026-12796-4

**Published:** 2026-04-02

**Authors:** Marios Alogakos, Ahmed Ghani, Abdullah R. Ayesh, May Y. Hajeir, Lamees M. Al Darwashi, Christian A. Than, Hayato Nakanishi, Benjamin E. Johnson, Alexandra M. Roch, Eugene P. Ceppa

**Affiliations:** 1 Department of Surgery, Division of Surgical Oncology, Indiana University School of Medicine, 545 Barnhill Drive, EH 541, Indianapolis, IN 46202 USA; 2 School of Medicine, University College Dublin, Dublin, D04 C1P1 Ireland; 3 School of Medicine, University of Nicosia, 2417 Nicosia, Cyprus; 4 Cambridge University Hospitals NHS Foundation Trust, Addenbrookes Hospital, Cambridge, CB2 0QQ UK; 5 School of Biomedical Sciences, The University of Queensland, St Lucia, 4072 Australia; 6 Department of Surgery, Mayo Clinic, Rochester, MN 55905 USA; 7 Department of Surgery, Endeavor Health, Chicago, IL 60625 USA

**Correction to: Surgical Endoscopy** 10.1007/s00464-026-12715-7.

The original online version of this article was revised to correct a typographical error in the abstract. The corrected text reads: The overall postoperative morbidity was 16.2% (95% CI 0.092–0.233, *I*^2^ = 60%, *n* = 47).

The original article has been corrected.

