# Supplementary figures and images for: Minimally invasive repair of iatrogenic bile duct injuries: a systematic review and meta-analysis
Source: Surg Endosc. 2026 Mar 30;40(5):3609–27. doi: 10.1007/s00464-026-12715-7 (PMC13161001; doi:10.1007/s00464-026-12715-7)

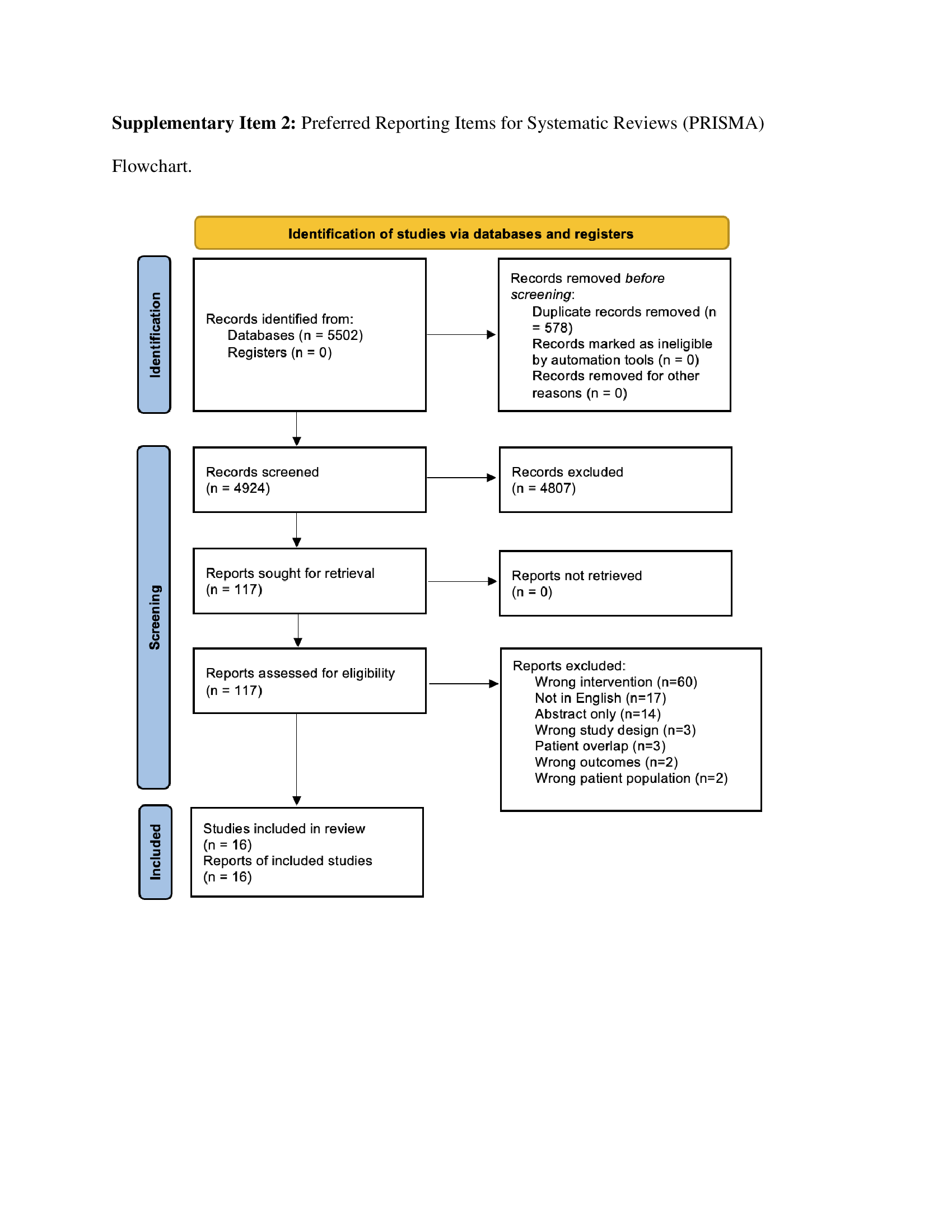

Supplement: Supplementary file 2 — Supplementary file1 (TIFF 270 KB) [file 464_2026_12715_MOESM2_ESM.tiff]

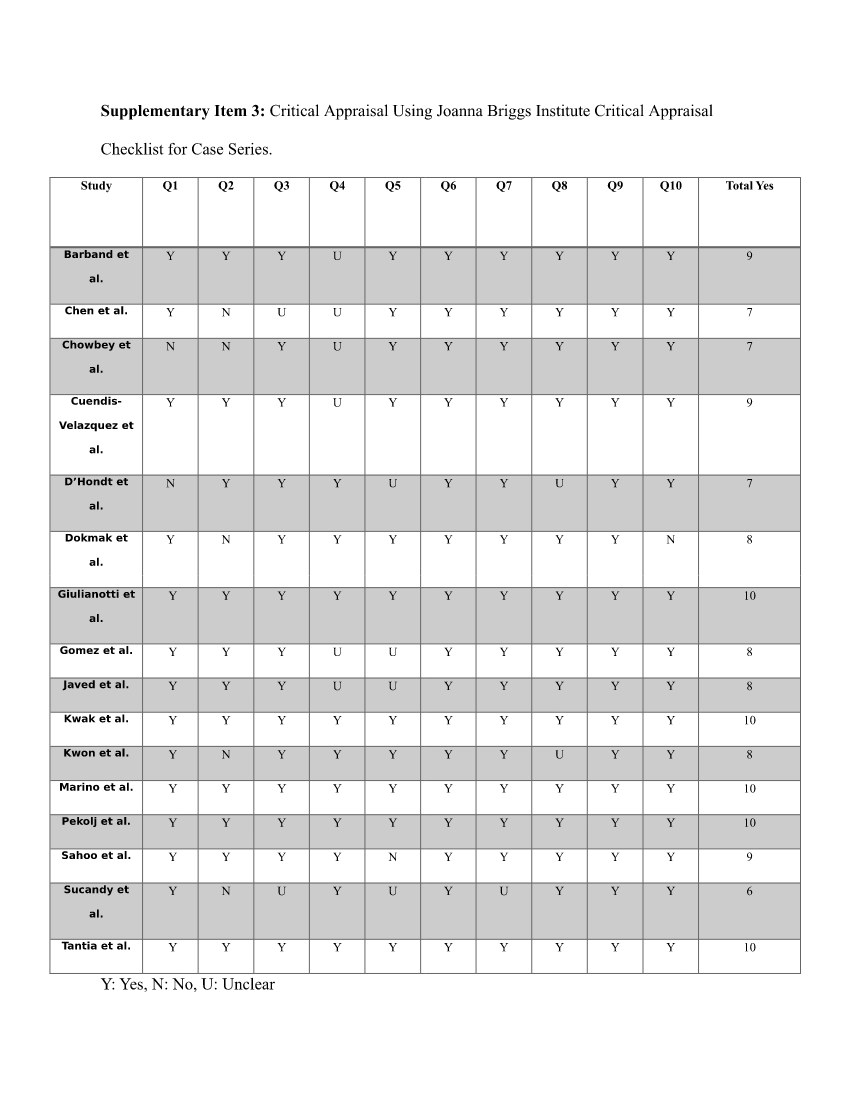

Supplement: Supplementary file 3 — Supplementary file1 (TIFF 3656 KB) [file 464_2026_12715_MOESM3_ESM.tiff]

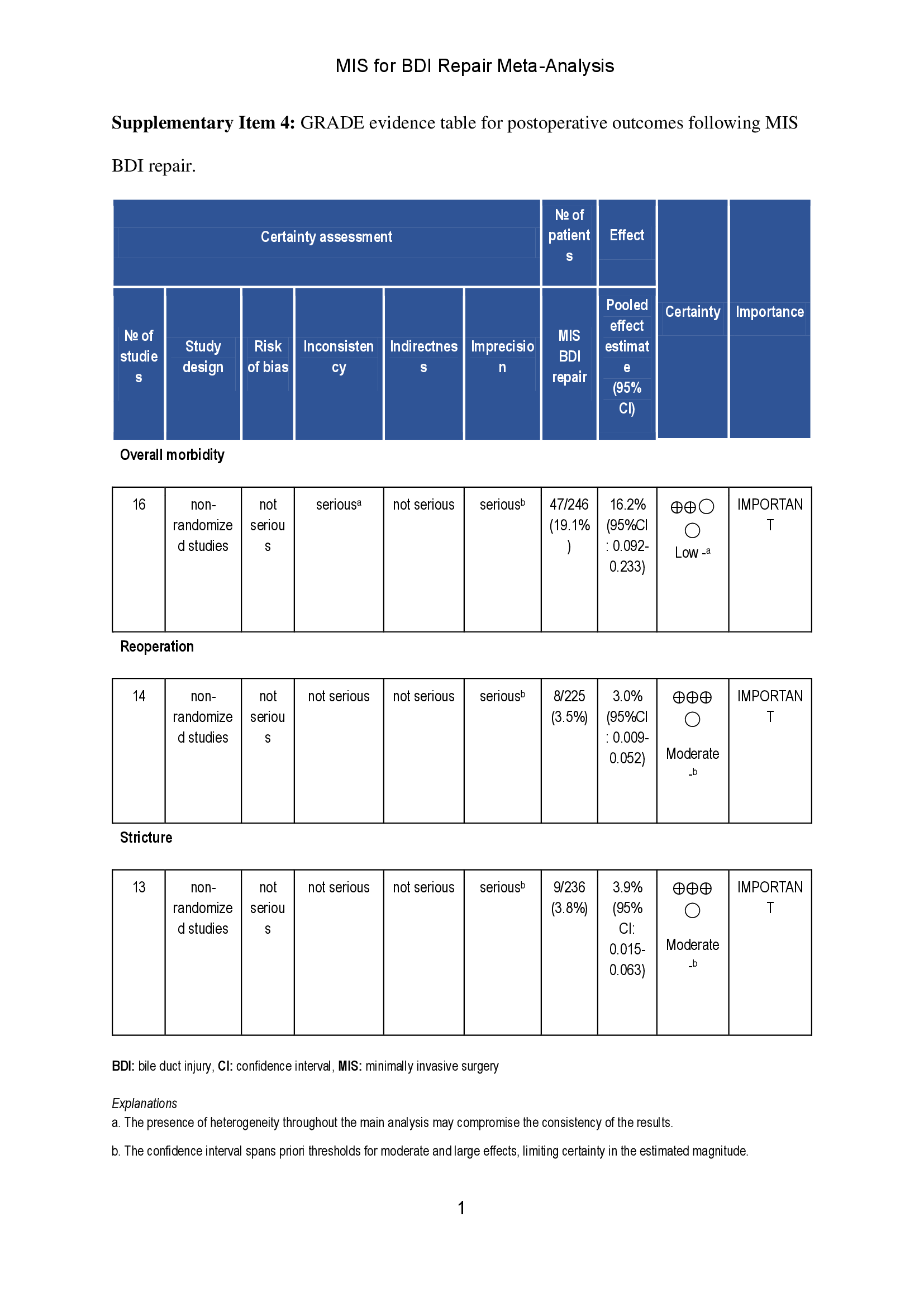

Supplement: Supplementary file 4 — Supplementary file1 (TIFF 241 KB) [file 464_2026_12715_MOESM4_ESM.tiff]
